# Research on the Oxidation Mechanism of Vermicular Graphite Cast Iron

**DOI:** 10.3390/ma12193130

**Published:** 2019-09-25

**Authors:** Qiaoqin Guo, Zhong Yang, Ding Guo, Dong Tao, Yongchun Guo, Jianping Li, Yaping Bai

**Affiliations:** 1 School of material and chemical engineering, Xi’an Technological University, Xi’an 710032, China; yz750925@163.com (Z.Y.); lunwentaodong@163.com (D.T.); yc_guo@163.com (Y.G.); jpli0416@yahoo.com.cn (J.L.); jingpingxue2004@163.com (Y.B.); 2 Xi’an Capital Water Limited Company, Xi’an 710086, China; guoding0223@163.com

**Keywords:** oxidation mechanism, vermicular graphite iron, oxidation channel, graphite, scanning electron microscope (SEM)

## Abstract

The oxidation mechanism of vermicular graphite cast iron was studied. The oxidation reaction starts from graphites and diffused slowly. Graphites in vermicular graphite are interconnected, coral-like clusters, providing the main oxidation core and channel. The worm-like graphites on the surface are mostly oxidized and form oxide affected zones. The oxide films are composed of a loose oxide layer with the phases of Fe_3_O_4_, Fe_2_O_3_, and FeO, and a dense passivation layer with FeO and Fe_2_SiO_4_. After oxidation, pearlites in the vermicular graphite cast iron are decomposed into ferrite and cementite at high temperatures.

## 1. Introduction

Vermicular graphite cast iron is a kind of transition state cast iron with transition forms between flake and spheroidal graphite. It is a new engineering material with fine mechanical properties, thermal conductivity, and section conductivity. Its strength, plasticity, and toughness are superior to that of gray iron. Its castability, thermal fatigue properties, and heat conductivity are better than those of ductile iron [1,2,3]. Therefore, vermicular graphite cast iron has been widely used in cylinder heads and engine blocks internationally. However, with the improvement of diesel engine performance and the increase of specific power, the peak fuel ignition pressure in the cavity needs to be increased, which leads to the increase of engine cylinder block and cylinder head operating temperatures [4].

However, at high temperatures, vermicular iron is badly oxidized, sometimes causing serious economic loss and security risks. Its application conditions are more and more severe with the rapid development of modern industry. Oxidation has become a significant apparent danger in the application of vermicular iron. According to Ref. [5], the author found that graphite size and quantity had an important influence on oxidation in cast iron. In [6], the authors studied the effects of chromium, molybdenum, aluminum, and other alloying elements on the oxidative performance of vermicular iron. In Ref. [7], the effect of the percentage of vermicular graphite on the oxidative performance of vermicular graphite cast iron was studied. In Ref. [8,9], surface treatment methods such as multi-arc and magnetron sputtering ion were used, which can improve oxidation resistance, although the improvement level is limited. The study of oxidation layer growth and the internal three-dimensional graphite structure are the keys for studying the oxidation-resistance and surface treatment of vermicular graphite cast iron; neither avenue has been explored systematically. In this paper, the oxidation mechanism of vermicular graphite cast iron is studied in order to lay a foundation for theory and practice. 

## 2. Experimental 

The compositions of vermicular graphite cast iron were C (wt%): 3.8%, Si (wt%): 2.1%, Mn (wt%): <0.2%, Mo (wt%): 0.2%, S (wt%): <0.03%, P (wt%): <0.05%, Fe (wt%): residual.

Mg-Re alloys were the vermiculizer. Their compositions were Re (wt%): 14%, Si (wt%): 39%, Fe (wt%): 41.9%, Mg (wt%): 2.5%, Ca (wt%): 1.9%.

Since the work temperature of a cylinder head is 530 °C, in order to be close the operating conditions, the oxidation experiment was performed in a muffle furnace set at 530 °C. The size of the test bars was Φ15 mm × 30 mm; they were washed in acetone. The microstructure of the vermicular graphite cast iron was observed via JSM-6700F scanning electron microscopy (SEM, JEOL, Tokyo, Japan). The phase compositions of the oxide films were tested via XRD-7000S X-ray diffraction (XRD) diffractometer (Shimazu, Kyoto, Japan). Three-dimensional XRT morphology of graphite in vermicular graphite was determined by Xradia Versa XRM-500 (Zeiss, Jena, Germany). 

## 3. Results and Discussions

**Figure 1:**
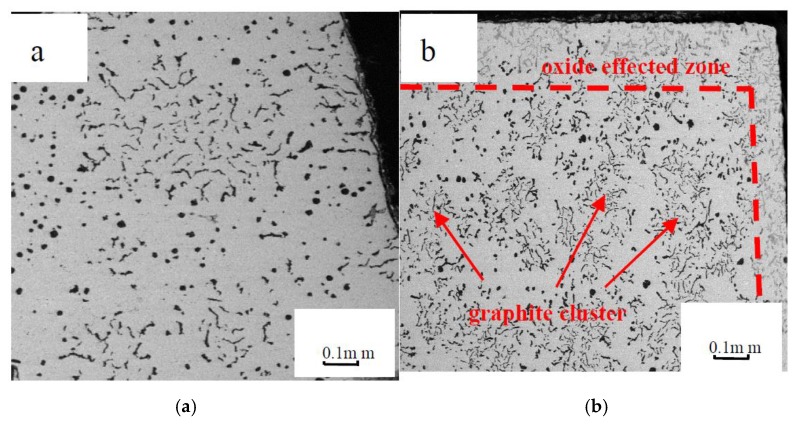
The surface morphology of vermicular graphite iron (vermicular graphite rate is 89%). (**a**) Before oxidation; (**b**) after oxidation for 500 h.

Figure 1 shows the morphologies of vermicular graphite cast iron before and after oxidation.

Before oxidation, when the vermicular rate was 89%, the surface of vermicular graphite cast iron was composed of vermicular graphite and spherical graphite without oxidation, as shown in Figure 1a. However, after oxidation for 500 h, as shown in Figure 1b, the worm-like graphites on the surface of the material were mostly oxidized, and oxide affected zones appeared with serious oxidation. In the center, the worm-like graphite clusters were oxidized and nodular graphites were not oxidized, demonstrating that the worm-like graphites corrode easily, in contrast to nodular graphites.

**Figure 2:**
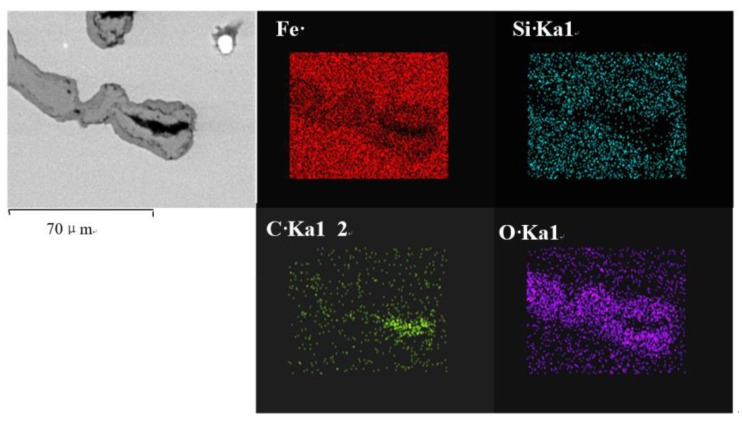
Analysis of area distribution of elements.

An analysis of element area distributions is shown in Figure 2. 

**Figure 3:**
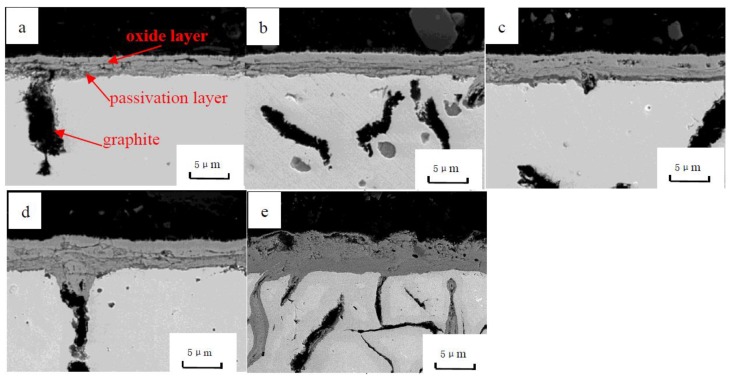
The cross-section morphologies of vermicular graphite cast iron at different oxidation times. (**a**) 100 h; (**b**) 200 h; (**c**) 300 h; (**d**) 400 h; (**e**) 500 h.

In order to determine the growth process of the oxidation layer, the cross-section morphologies of vermicular graphite cast iron at different oxidation times were observed, as shown in Figure 3. It is clear that the oxidation layers grow gradually, and that their thickness increase with oxidation time.

**Figure 4:**
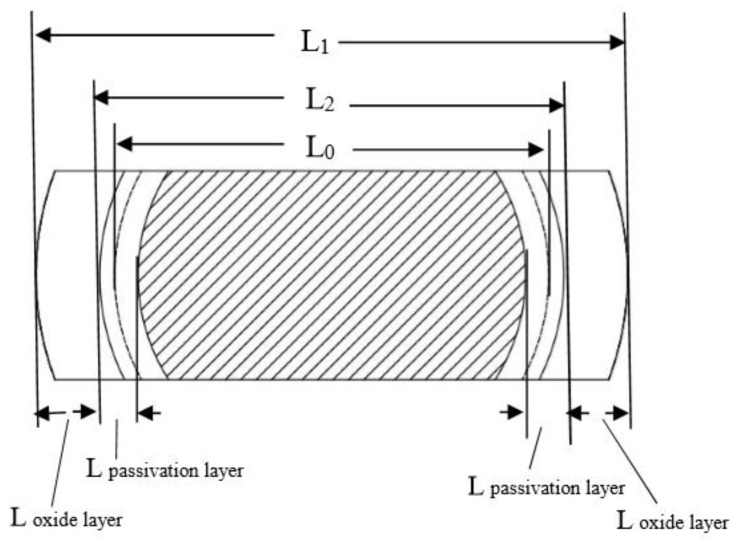
Parameters of oxide film.

When vermicular iron was oxidized for 500 h, the oxidation layer thickness reached about 40 μ m. As shown in Figure 3a–c, in the first 300 h, the oxidation was mainly concentrated on the surface of vermicular graphite cast iron. But there was less oxidation in graphite. As the oxidation goes on, the matrix is oxidized, depending on graphite as the oxidation channel, and its oxidation products increase. The oxidation layers are composed an oxide and a passivation layer. With the extension of the oxidation time, the oxide layer and the passivation layer are continuously thickened, but the interface distance (L_2_) of the passivation layer is slightly increased compared with the original size (L_0_) of the sample, as shown in Figure 4 (the increase of this size is related to the phase transition in the passivation layer). This indicates that:

(a) The cross section of the passivation layer and the oxidation layer does not migrate significantly with the extension of time, so it can be considered that this cross section is the original surface before oxidation.

(b) The oxide film grows outwards and inwards simultaneously, with the oxide layer growing outwards and the passive layer growing inwards. This is because the Fe^2+^ diffuses to the outside, being oxidized to form Fe_x_O_y_ on the interface between the oxide film and the atmosphere, which leads to the oxide film growing outwards gradually and the size of the sample increasing. Meanwhile, as O^2−^ diffuses inward through the oxide layer and Fe^2+^ and Si^4+^ diffuse outward, FeO and Fe_2_SiO_4_ are generated at the metal-oxide film interface, so the passivation layer can gradually grow inward [10,11,12,13].

It can be seen from Figure 3, in the cross section, that the oxidation channels are graphites. When the vermicular graphite iron is oxidized, the oxygen diffusion rate is determined by the matrix continuity. When graphites exist, the interface pores between graphites and matrix are reduced. The rate of oxygen passing through the interface became greater than that of penetration through the matrix [14,15,16]. Some graphites are hollow after decarburization, leading an increase in the likelihood of the spread of oxygen. 

**Figure 5:**
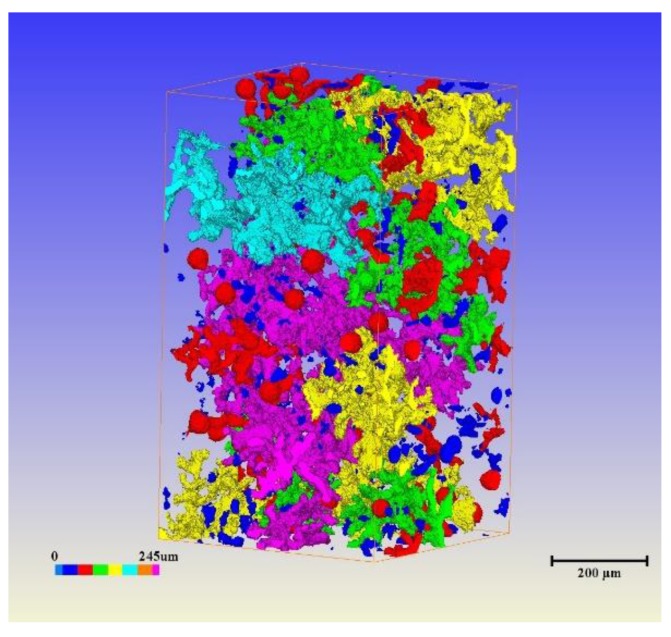
Three-dimensional morphology of graphite in vermicular graphite by XRT. (with 89% vermicular graphite rate).

Figure 5 shows that the 3-dimensional structure of graphite in the vermicular iron by XRT comprises interconnected coral-like clusters. The 3-dimensional morphology of vermicular graphite is obviously different from that of 2-dimensional graphite, and the coral-like clusters are obvious, especially for graphite with large connections. 

Except for some small graphites, the volumes of individual coral graphites are difficult to detect, and the 2-dimensional structure of an isolated graphite eutectic cell form is difficult to evaluate in 3- dimensional structure. Therefore, the greater the interface area, the greater the diffusion rate of oxygen, leading to a high oxidation rate [17,18,19,20]. When graphites exit in different shapes, the surface areas of the substrate and graphite vary greatly. As for the same volume of graphite, when the graphite morphologies are respectively globular, vermicular, and flake, the interface areas vary from small to large. The rate of oxygen permeation into the inner layer of globular, vermicular, and flake graphite cast iron gradually increases [21,22,23,24,25]. The oxidation rate similarly increases. The thicknesses of the oxidization layer are also th gradually increased ickened. 

**Figure 6:**
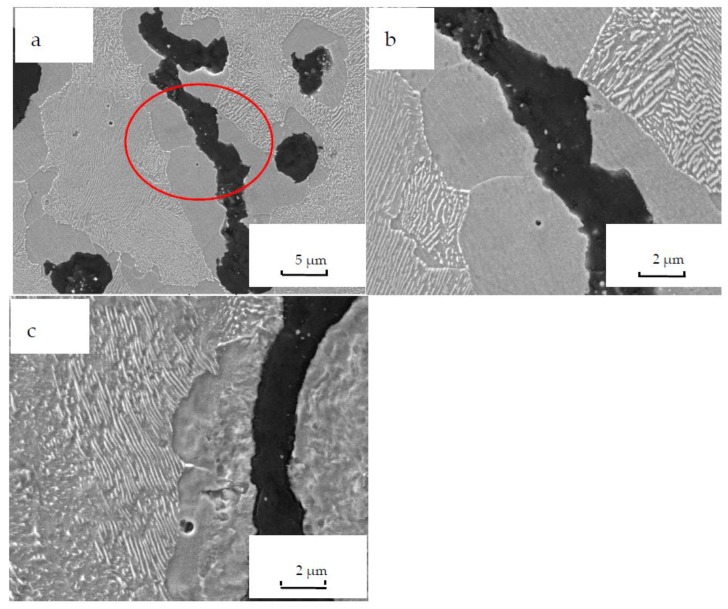
Microstructures of vermicular iron (with etching) (**a**) oxidation for 0 h, 2000×, (**b**) oxidation for 0 h, 5000×, and (**c**) oxidation for 500 h, 5000×.

Figure 6 shows the morphologies of vermicular graphite cast iron before and after oxidation. It can be seen that they comprise a matrix with graphite, ferrite, and pearlite. The amount of ferrites is less than that of pearlites before oxidation, as shown in Figure 6a. However, Figure 6c shows that after oxidation, the ferrites are much more prevalent than pearlites after oxidation. The reason for this is that pearlites are decomposed into ferrite and cementite after exposure to high temperatures for long periods.

Meanwhile, concerning graphite, much more oxidation product appears, as shown in Figure 6c. As discussed in Figure 3, there are interfaces between graphite and the matrix. Oxygen easily enters the matrix and diffuses, so oxidation product can be seen around graphite. 

**Figure 7:**
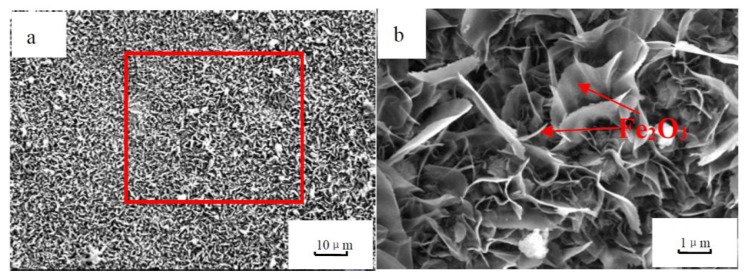
Surface morphology oxidized at 530 °C for 500 h (**a**) 1000×, (**b**) 5000×.

Figure 7 shows the surface microstructures of vermicular iron. It can be seen that there are dense oxide films with needle-like and flake shapes on the surface of vermicular iron. According to Ref. [26], Fe_2_O_3_ is dense and exhibits flakes and needle-like structures. Fe_3_O_4_ shows a grain-shape, but is difficult to observe on the surface microstructure since it is dispersed under the needle-shapes and flakes of Fe_2_O_3_.

**Figure 8:**
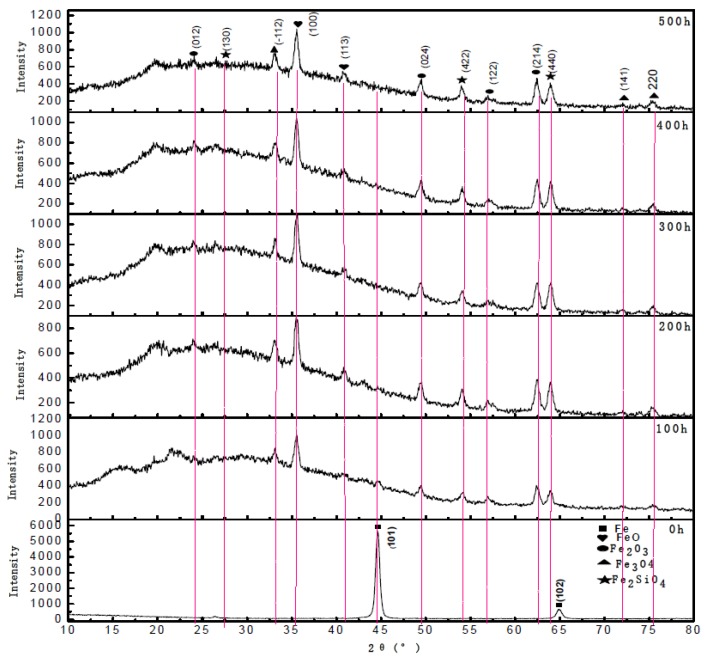
XRD spectra of vermicular iron oxidized for different time.

The phase compositions of vermicular iron were studied at different times, as shown in Figure 8. It is clear that the vermicular iron matrix grains grow along Fe (102) and Fe (101), and that the intensity of peak Fe (101) is considerably higher than that of peak Fe (102). The Fe phase disappears after oxidation for 500 h and the surface is covered by thick oxide films with FeO, Fe_2_O_3_, Fe_3_O_4_, and Fe_2_SiO_4_. The oxidation reactions are as follows: 2Fe + O_2_ = 2FeO, 3Fe + 2O_2_ = Fe_3_O _4_, and FeO + O_2_ = Fe_2_O_3_. 2Fe + O_2_ + SiO_2_ = Fe_2_SiO_4_. The inside closing to the vermicular iron matrix is the passivation layer with dense FeO and Fe_2_SiO_4_ [27,28,29,30,31]. 

## 4. Conclusions

The oxidation mechanism of vermicular graphite cast iron was studied in this paper. The oxidation reaction started from graphite and diffused slowly to the Fe substrate. The worm-like graphites on the surface were mostly oxidized, and there were oxide affected zones with serious oxidation from the edge to the center. The oxidation reaction started from graphites and diffused slowly. From the exterior to the interior, the oxide layers were mostly composed of Fe_3_O_4_, Fe_2_O_3_, and FeO, and a passivation layer with FeO and Fe_2_SiO_4_. Graphites in vermicular graphite are interconnected, coral-like clusters, providing the oxygen channel. After oxidation, pearlites are decomposed into ferrite and cementite at high temperatures. Meanwhile, regarding graphite, many more oxidation products appeared.
